# *Leptomonas seymouri*: Adaptations to the Dixenous Life Cycle Analyzed by Genome Sequencing, Transcriptome Profiling and Co-infection with *Leishmania donovani*

**DOI:** 10.1371/journal.ppat.1005127

**Published:** 2015-08-28

**Authors:** Natalya Kraeva, Anzhelika Butenko, Jana Hlaváčová, Alexei Kostygov, Jitka Myškova, Danyil Grybchuk, Tereza Leštinová, Jan Votýpka, Petr Volf, Fred Opperdoes, Pavel Flegontov, Julius Lukeš, Vyacheslav Yurchenko

**Affiliations:** 1 Life Science Research Centre, Faculty of Science, University of Ostrava, Ostrava, Czech Republic; 2 Department of Parasitology, Faculty of Science, Charles University, Prague, Czech Republic; 3 Zoological Institute of the Russian Academy of Sciences, St. Petersburg, Russia; 4 Biology Centre, Institute of Parasitology, Czech Academy of Sciences, České Budějovice (Budweis), Czech Republic; 5 de Duve Institute and Université catholique de Louvain, Brussels, Belgium; 6 Faculty of Sciences, University of South Bohemia, České Budějovice (Budweis), Czech Republic; 7 Canadian Institute for Advanced Research, Toronto, Ontario, Canada; 8 Department of Pathology, Albert Einstein College of Medicine, Bronx, New York, United States of America; Institut de Recherche pour le Développement (IRD), FRANCE

## Abstract

The co-infection cases involving dixenous *Leishmania* spp. (mostly of the *L*. *donovani* complex) and presumably monoxenous trypanosomatids in immunocompromised mammalian hosts including humans are well documented. The main opportunistic parasite has been identified as *Leptomonas seymouri* of the sub-family Leishmaniinae. The molecular mechanisms allowing a parasite of insects to withstand elevated temperature and substantially different conditions of vertebrate tissues are not understood. Here we demonstrate that *L*. *seymouri* is well adapted for the environment of the warm-blooded host. We sequenced the genome and compared the whole transcriptome profiles of this species cultivated at low and high temperatures (mimicking the vector and the vertebrate host, respectively) and identified genes and pathways differentially expressed under these experimental conditions. Moreover, *Leptomonas seymouri* was found to persist for several days in two species of *Phlebotomus* spp. implicated in *Leishmania donovani* transmission. Despite of all these adaptations, *L*. *seymouri* remains a predominantly monoxenous species not capable of infecting vertebrate cells under normal conditions.

## Author Summary

In this work we performed a comprehensive evaluation of the infective potential of *Leptomonas seymouri*, repeatedly isolated from kala-azar patients infected by *Leishmania donovani* in India and neighboring countries, and have tested the capacity of this monoxenous trypanosomatid to utilize the sand fly vectors permissive for *Leishmania donovani*. We concluded that despite several genetic adaptations it has developed, *Leptomonas seymouri* remains a predominantly monoxenous species not able to infect mammalian macrophages either alone or in co-infection with *Leishmania*. Under certain circumstances it is able to infect mammals, but probably only when the host is immunocompromised by infection with another pathogen, such as *Leishmania donovani* or HIV.

## Introduction

Flagellates of the family Trypanosomatidae are single-celled obligatory parasites. They can be either dixenous (i.e. those with two hosts in their life cycle—*Trypanosoma*, *Leishmania*, and *Phytomonas* spp.) or monoxenous (i.e. those having only one host). For decades, monoxenous trypanosomatids of insects were effectively neglected. However, this situation is rapidly changing, as a remarkable diversity of these flagellates is being revealed within insects—a group which is known to be extraordinarily species rich [1,2]. In addition, the study of these parasites is expected to shed light on the origin of the dixenous life cycle (alternation of an insect vector and a vertebrate or plant host). It is generally accepted that the dixenous species have evolved from their monoxenous kins and that this transition has happened independently at least three times during the evolution of Trypanosomatidae, as the dixenous genera *Trypanosoma*, *Leishmania*, and *Phytomonas* are interspersed by the monoxenous genera *Angomonas*, *Blastocrithidia*, *Blechomonas*, *Crithidia*, *Herpetomonas*, *Kentomonas*, *Leptomonas*, *Paratrypanosoma*, *Sergeia*, *Strigomonas*, and *Wallacemonas* (S1 Fig) [3,4]. This suggests that some (presumably) monoxenous species may occasionally try switching to dixeny. Indeed, the presence of the monoxenous trypanosomatids in vertebrates has been noted already about 100 years ago [5]. More recently, several monoxenous flagellates belonging to the genera *Herpetomonas*, *Crithidia*, *Leptomonas*, and *Blechomonas* have been identified from human clinical isolates [6–8]. Importantly, most of them involved immuno-compromised individuals, leading to a hypothesis that these usually non-infectious species may explore new ecological niches in vertebrates that have their immune system suppressed [9,10]. Within this paradigm, about two dozen cases of monoxenous trypanosomatids co-infecting humans along with various *Leishmania* spp. have been reported almost exclusively from the Indian subcontinent. Most of them implicated causative agents of visceral leishmaniasis (kala-azar) of the *L*. *donovani* complex [11]. It was also demonstrated that both dixenous and monoxenous flagellates may be transmitted by the same *Phlebotomus* vector, yet the evidence is not very strong [12,13]. The cytochrome b and 18S rRNA-based PCR analyses were confined to the isolates from a small geographical area and the identity of non-*Leishmania* parasites could not be elucidated to the species level.

The species most often recovered from co-infections in leishmaniasis patients is *Leptomonas seymouri* Wallace, 1959 [14]. Together with all *Leishmania* spp. it belongs to the subfamily Leishmaniinae (S1 Fig) [15] and was originally isolated from a cotton stainer *Dysdercus suturellus* (Hemiptera: Pyrrhocoridae) [16]. Nonetheless, when a broad-scale survey of trypanosomatids parasitizing pyrrhocorids throughout the world was undertaken, none of the samples proved to contain *L*. *seymouri* [17]. So the question remains whether the original isolate was obtained from a specific host (e.g. species that is evolutionary adapted for parasite's life cycle). *L*. *seymouri* can even multiply in plants under experimental conditions [18] proving it to be non-fastidious and able to adapt to quite different environments.

Recent whole-genome analysis of kala-azar clinical isolates from splenic aspirates demonstrated heavy "contamination" with unidentified *Leptomonas* sp. [19]. This result is not so surprising provided that both parasites are almost indistinguishable by morphology and that *Leptomonas* outgrows *Leishmania* in culture [20].

We speculate that several species of monoxenous trypanosomatids are capable of surviving in the hostile environment of the vertebrate body. Molecular details of such adaptation are not worked out, yet it is clear that some monoxenous trypanosomatids must be able to tolerate heat shock up to the temperatures they might experience in warm-blooded vertebrates. Indeed, a number of representatives of the genera *Crithidia* and *Herpetomonas* can withstand elevated temperature reaching 37°C [21–23].

In this study we addressed the issue of *Leishmania–Leptomonas* co-infection from the point of view of the monoxenous partner. To understand molecular mechanisms and biochemical pathways responsible for survival within warm-blooded vertebrates, we have demonstrated that *Leptomonas seymouri* can withstand elevated temperatures *in vitro*, sequenced its genome, and assessed transcriptional profiles of cells cultivated in different conditions. Furthermore, we tested *L*. *seymouri* ability to survive in *Phlebotomus argentipes* and *P*. *orientalis*, two sand fly species implicated in *Leishmania donovani* transmission.

## Results

### Identification of *Leptomonas seymouri* in clinical kala-azar isolates

Whole genome sequencing of two clinical Indian kala-azar field isolates, a strain resistant to sodium antimony gluconate therapy (Ld 39, May 2000, Muzaffarpur, Bihar) and a strain sensitive to treatment (Ld 2001, February 2000, Balia, Uttar Pradesh), revealed numerous (over 95%) sequences apparently derived from *Leptomonas* sp. in addition to those of *L*. *donovani* [19]. These isolates were cultivated from splenic aspirates in frame of a large screen aimed to understand molecular differences between confirmed kala-azar cases. For precise identification of the co-infecting species we applied an arsenal of molecular tools developed over the years [24–27]. Three genetic loci, namely 18S rRNA, glycosomal glyceraldehyde-3-phosphate dehydrogenase (gGAPDH), and ITS regions were amplified, sequenced and compared with other representatives of the subfamily Leishmaniinae [15]. 18S rRNA sequences of the isolates Ld 39 and Ld 2001 (GenBank accession numbers KP717894 and KP717895, respectively) were identical and indistinguishable from the corresponding sequence of *L*. *seymouri* (GenBank accession number AF153040). gGAPDH sequences (GenBank accession numbers KP717896 and KP717897 for isolates Ld 39 and Ld 2001, respectively) were nearly identical with only 1 nt substitution in the coding sequence. They both were very similar (except for the degenerative primer sequences) to the gGAPDH sequence of *L*. *seymouri* (GenBank accession number AF047495). 18S rRNA and gGAPDH sequences are informative for higher level taxonomy, and are usually adequate for the genus (and up) level ranking [4,28]. For proper species identification we used other well-established markers, ITS1 and ITS2 [14,20,29]. Their sequences were identical with the exception of a 2 nt-long indel (GenBank accession numbers KP717898 and KP717899 for isolates Ld 39 and Ld 2001, respectively). BLAST search revealed 100% identity with the ITS1-5.8S rRNA region of *L*. *seymouri* (GenBank accession number JN848802).

The data presented above allowed us to conclude that the monoxenous co-infectant of the clinical kala-azar isolates Ld 39 and Ld 2001 is *L*. *seymouri*. We also would like to note that the cases of co-infections of *Leishmania* and *Leptomonas* are likely underreported in the literature, as several sequences attributed to *L*. *donovani* in GenBank do in fact belong to *L*. *seymouri*. Our analysis of the ITS-containing region, SL, gGAPDH, HSP70, HSP83, RNA polymerase II, α-tubulin and some mitochondrial genes (A6, cytb, COI, COII, COIII, NADH) revealed that 38 out of 170 (22%) and 3 out of 217 (1.4%) ITS sequences of *L*. *seymouri* were misidentified as *Leishmania donovani* and *L*. *tropica*, respectively (see S1 Table for GenBank accession numbers).

### *Leptomonas seymouri* withstands elevated temperature typically associated with vertebrate infection

**Fig 1:**
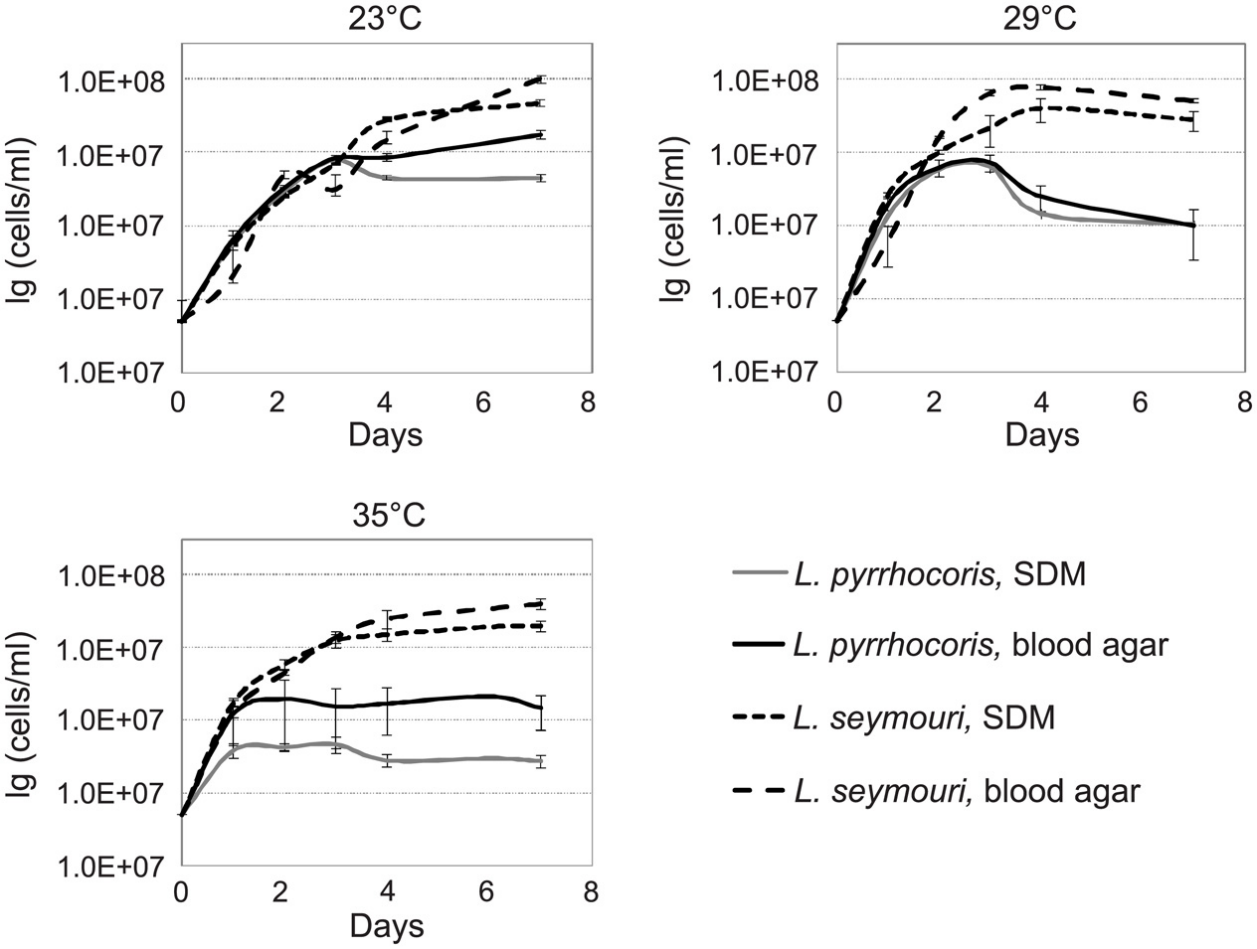
Growth kinetics of *Leptomonas pyrrhocoris* and *L*. *seymouri* at 23°C, 29°C, and 35°C in SDM and blood-agar media. Data from 3 independent biological replicates are presented.

The presence of monoxenous *L*. *seymouri* in co-infections with dixenous *L*. *donovani* implies several adaptations to the environment of the human body. One of the important factors to be considered is temperature. Typical monoxenous trypanosomatids of the insect gut are temperature-sensitive and cannot withstand conditions of the warm-blooded vertebrates [6]. In order to investigate temperature resistance of several trypanosomatid species *in vitro*, we compared growth kinetics of two different *Leptomonas* species, *L*. *seymouri* ATCC 30220 (hereafter used as a proxy of filed isolated Ld 39 and Ld 2001, which were not available) and *L*. *pyrrhocoris* H10, under different experimental conditions. Parasites were incubated at temperatures 23°C, 29°C, and 35°C for up to 7 days. The highest temperature (35°C) approximately corresponds to that faced by the flagellates upon transfer from a sand fly into a vertebrate. To imitate the conditions of insect gut and vertebrate blood, SDM and two-phased blood-agar were used, respectively. No considerable difference was observed in growth kinetics of two trypanosomatid species incubated at 23°C in both media. Interestingly, increasing the cultivation temperature to 29°C and 35°C inhibited growth of *L*. *pyrrhocoris*, while growth of *L*. *seymouri* was not significantly affected (Fig 1). We concluded that *L*. *seymouri* is capable of withstanding the elevated temperature reaching that of the human body. In contrast, *L*. *pyrrhocoris* is temperature-sensitive and halts its cell division in non-optimal conditions. In all cases, cultivation on blood-agar medium resulted in higher cells density.

**Fig 2:**
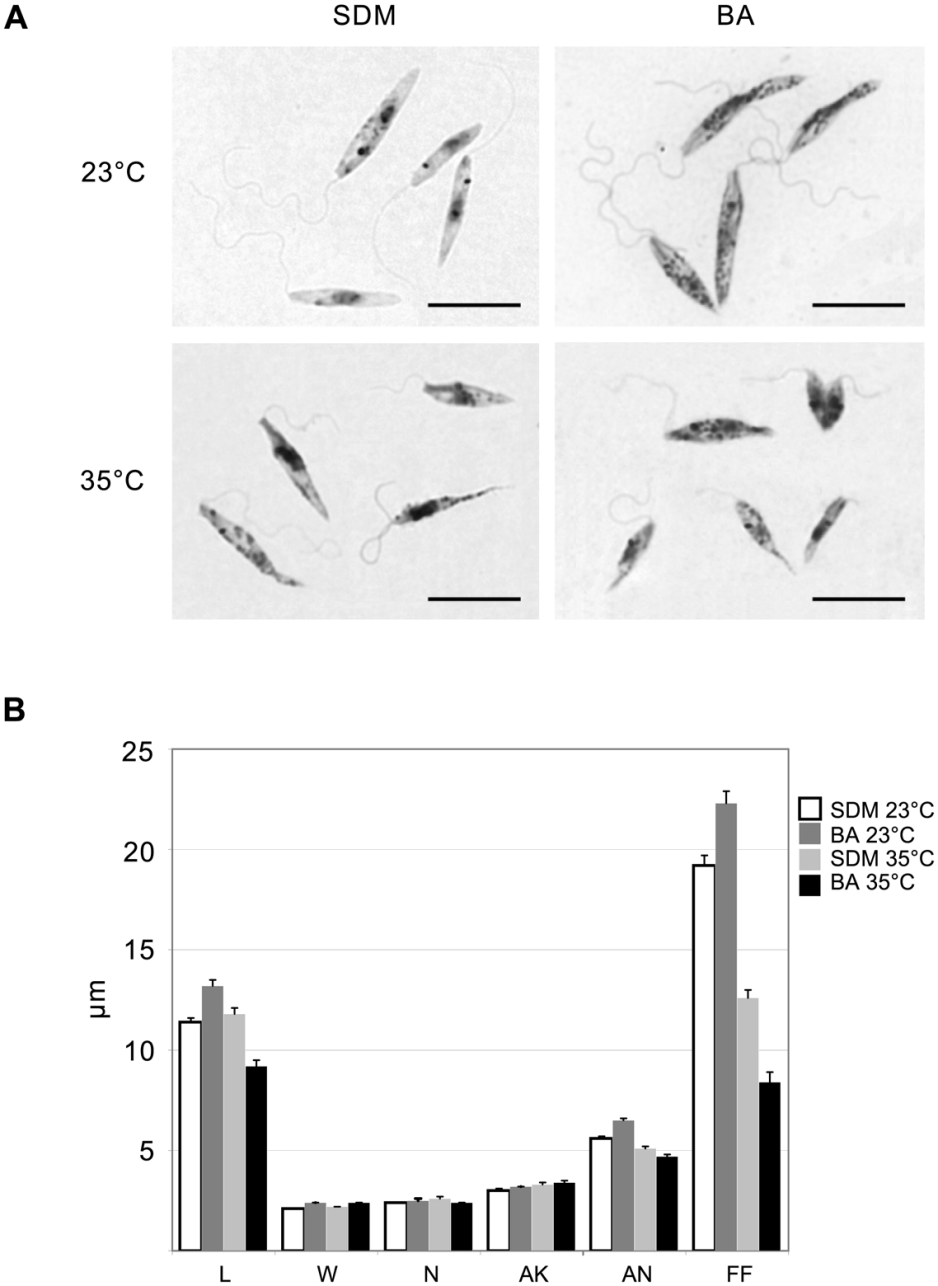
Morphology of *Leptomonas seymouri* cells cultivated *in vitro* in SDM and blood-agar media at low (23°C) and high (35°C) temperature. **A**, Giemsa-stained slides, scale bar is 10 μm. **B**, ANOVA statistical analysis of 100 cells, average and standard error are presented in micrometers (μm). L–length, W–width, N–length of the nucleus, AK–distance between the kinetoplast and anterior end of the cell, AN–distance between the nucleus and anterior end of the cell, FF–free flagellum length.

Light microscopy of Giemsa stained smears of *L*. *seymouri* cultivated under different experimental conditions revealed statistically significant morphological changes (Fig 2). The most noticeable one was shortening of the free portion of the flagellum observed in cells cultivated at high temperature. This phenomenon was observed for both media used but it was more pronounced in blood-agar. Also elevated temperature resulted in more diverse body sizes and shapes with the most conspicuous feature being elongated and tapered posterior end of some cells.

### Genome of *Leptomonas seymouri*

The genome of *L*. *seymouri* ATCC 30220 was assembled into 1,222 scaffolds (maximum length 326,845 bp) with N50 of 70,646 bp and a total assembly length of approximately 27.3 Mbp. This is a substantial improvement over the previously reported assembly of the unidentified *Leptomonas* sp. (14,518 contigs with maximum length of 26,366 and N50 of 3,370 bp) [19]. Both assemblies had almost the same total genome length (27.3 and 27.4 Mb). Importantly, over 85% of the reads could be cross-mapped (length fraction = 0.9; similarity fraction = 0.9) confirming identity of the *L*. *seymouri* isolates. The number of annotated protein-coding genes, 8,488, was also within the range of previously reported genomes (6,451 for *Phytomonas* sp. HART1; 8,309 for *Leishmania major*; 10,109 for *Trypanosoma brucei*) [30–32]. Consistent with other trypanosomatids, the protein-coding genes lack conventional introns. The only exceptions reported so far in *Trypanosoma* spp. and *Leishmania* spp. are poly(A) polymerase and DEAD/H RNA helicase [32,33]. Indeed, their *L*. *seymouri* orthologs also contain introns and thus require *cis-*splicing for proper expression.

A typical aspect of the *L*. *seymouri* genome is that it contains a relatively small number of genes that have undergone tandemly linked duplications. Using a cutoff value of 10^−50^, the number of genes present in two or more homologous copies has been estimated at about 9.9% in *L*. *seymouri*. Same numbers for *Phytomonas* sp., *L*. *major*, *T*. *brucei*, and *C*. *fasciculata* are 9.6%, 18.3%, 26.0%, and 40.2%, respectively. This is one of the major components determining differences in genome size among these species.

### Metabolic pathways in *L*. *seymouri*

Genomic information was used to predict the metabolic pathways in *L*. *pyrrhocoris* and *L*. *seymouri*, two phylogenetic kins with different sensitivity to temperature and ability to co-infect vertebrate hosts (S2 Fig). In essence, the metabolism in these two species is very similar, with important features and differences highlighted below. A classical glycolytic pathway, partly inside glycosomes (as inferred from the presence of peroxisome targeting signals), is responsible for the metabolism of various exogenous sugars (S2 Table). Carbohydrate metabolism is characterized by an incomplete aerobic oxidation because one of the classical mitochondrial tricarboxylic acids (TCA) cycle enzymes (NAD-linked isocitrate dehydrogenase) is absent. However, the other TCA cycle enzymes can be used for the inter-conversion of metabolic building blocks required for gluconeogenesis and other biosynthetic purposes (S3 Table). While both *L*. *pyrrhocoris* and *L*. *seymouri* are able to synthesize their own pyrimidines, they depend on a supply of external purines. They lack the capacity to oxidize aromatic amino acids and require an external supply of most of the essential amino acids, cofactors and vitamins for growth (S4 Table). Both *Leptomonas* spp. have a fully developed mitochondrion with 9 of the 10 TCA cycle enzymes present, a complete respiratory chain with the respiratory complexes I—IV, and a fully functional mitochondrial F_1_-ATPase (S5 Table).

Although lacking the alternative oxidase found in many other trypanosomatids, *L*. *seymouri* possesses an alternative NADH dehydrogenase gene. Our analysis predicts that it is able to feed on a large variety of polysaccharides, carbohydrates, both hexoses and pentoses, with the anticipated end products of carbohydrate metabolism being acetate, succinate, carbon dioxide, ethanol, alanine, and D-lactate. *L*. *seymouri* has a complete set of β-oxidation enzymes, which are associated with the mitochondrion. A few additional lipid-metabolizing enzymes are present in the glycosomes. It appears that the analyzed flagellate does not possess a type-I system of fatty acid synthesis, but makes its fatty acids in the cytosol by the action of a series of elongases (S6 Table). It is able to oxidize 16 of the 20 amino acids, but the necessary enzymes for the metabolism of lysine and the three aromatic amino acids (phenylalanine, tyrosine and tryptophan) are lacking. The urea cycle is not functional since two mitochondrial enzymes of the cycle are missing (S7 Table). The remaining three cytosolic enzymes have all been acquired by lateral gene transfer and allow arginine to be utilized in polyamine biosynthesis. Surface proteins, previously identified in *Trypanosoma*, *Leishmania* and *Crithidia* spp., have also been found in *Leptomonas* (S8 Table). Homologues of GP63, amastin, 3’-nucleotidase, integral membrane protein, prohibitin, membrane-bound acid phosphatases MBPA1 and MBPA2 and tartrate-sensitive acid phosphatase, but not oligosaccharyl transferase, are present. Protection against oxidative stress in monoxenous trypanosomatids differs from their dixenous kins. In addition to the trypanothione system and the presence of many homologues of tryparedoxins and peroxiredoxins, all monoxenous species analyzed thus far have a bacterial-type catalase acquired by lateral gene transfer (S9 Table).

Enzymes of the RNA interference pathway, namely the homologs of the Argonaute (AGO1) and the two dicer proteins (DCL1 and DCL2) were not detected in *L*. *seymouri* (S10 Table). Importantly, they were found in the genome of *L*. *pyrrhocoris* arguing that these two closely related species differ in their ability to regulate gene expression by RNA interference.

### Lateral gene transfer

In the evolution of Trypanosomatidae many events of lateral gene transfer (LGT) have taken place, since genes of bacterial origin are frequently encountered in all trypanosomatid lineages [34]. This suggests that an ancestral flagellate had already acquired such genes, which include a number of enzymes of glycolysis, pentose-phosphate shunt and pyrimidine biosynthesis, as well as trypanothione reductase and pterin transporters [35–37]. Some LGT events including genes involved in sucrose and pentose sugar metabolism, haem synthesis and urea cycle seem to be more recent and specific to the Leishmaniinae clade that comprises *Leishmania*, *Crithidia* and *Leptomonas* spp. [38–40]. Even more recent acquisitions, shared only among *Crithidia* spp. and *Leptomonas* spp. include catalase, the diaminopimelate-metabolizing enzymes and those of β-glucosidase, nitroalkane oxidase, phenolic acid dehydrogenase and glycerol dehydrogenase families (S11 Table). In total, 70 out of 586, or 12% of all the metabolic genes analyzed, have resulted from the events of lateral transfer.

### Gene family analysis using the OrthoMCL approach

For this analysis full proteomes for 23 trypanosomatid species were downloaded from TriTrypDB v. 7.0 and combined with newly annotated proteins from *L*. *seymouri*, *L*. *pyrrhocoris*, *B*. *ayalai* and *Paratrypanosoma confusum* (S12 Table). Comprehensive characterization of *L*. *seymouri* gene family repertoire and its comparison to that of other trypanosomatids may help to shed light on possible adaptations of this species to the dixenous lifestyle. Recently, a comparative genomics approach was used to define a "gene kit" implicated in cell invasion and intracellular parasitism in *Leishmania* spp. and *Trypanosoma cruzi* [41]. Authors have found that despite substantial differences in mechanisms of host cell invasion and survival within the host cell, 3,340 orthologous gene clusters are exclusively shared between intracellular parasites when compared to extracellular *T*. *brucei*. Many proteins within these clusters were already proven to play a pivotal role in *Leishmania* and *Trypanosoma* virulence (e.g. GP63, amastin, ascorbate peroxidase), while functions of other proteins require further detailed investigation.

In our study we were aiming to identify candidate proteins in *L*. *seymouri* that may define its ability to occasionally infect warm-blooded organisms. For that purpose Orthologous Groups (OG) presence/absence patterns in *L*. *seymouri* were analyzed and compared to those of other trypanosomatids. In the reference dataset for OrthoMCL analysis several *Leishmania* spp. (medically and veterinary important dixenous species), along with *C*. *fasciculata* and *L*. *pyrrhocoris* (both never encountered in vertebrates) are of primary interest for comparison with *L*. *seymouri*. According to a widely accepted view of trypanosomatid phylogeny, *Leptomonas* spp. are most closely related to *Crithidia* spp., and together they form a clade that clusters as a sister group to the genus *Leishmania* [2,15] (S2 Fig). Firstly, OG content was compared in *L*. *seymouri*, *C*. *fasciculata*, and *L*. *pyrrhocoris* in order to exclude from the analysis OGs that are present in typical monoxenous trypanosomatids. *Leptomonas pyrrhocoris* has a typical promastigote morphology and dwells in insect species of the family Pyrrhocoridae [17,42], while *C*. *fasciculata* uses various culicids as hosts [43–45]. Notably, some representatives of the genus *Crithidia* (*C*. *hutneri*, *C*. *luciliae thermophila*) can survive at temperatures of the mammalian and avian bodies [21,22]. Therefore *C*. *fasciculata* may possess genes involved in survival at elevated temperatures, and in order to exclude possible biases caused by the presence of *C*. *fasciculata* genes in our OrthoMCL analysis, OG repertoire comparisons were performed twice: with and without *C*. *fasciculata* in the datasets being compared.

**Fig 3:**
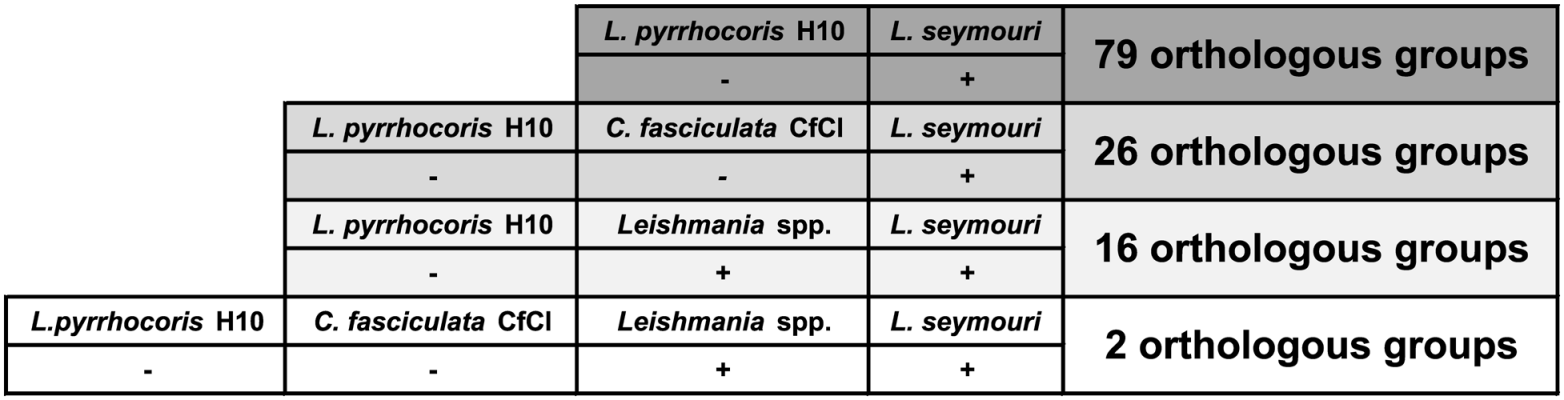
Orthologous group presence (denoted by "+")/absence (denoted by "-") patterns for *Leptomonas seymouri*, *Leptomonas pyrrhocoris*, *Crithidia fasciculata*, and several *Leishmania* species. The number of present OGs is indicated on the right.

Out of 7,935 *L*. *seymouri* OGs, 79 OGs were absent in *L*. *pyrrhocoris*, and 26 OGs were absent from both *L*. *pyrrhocoris* and *C*. *fasciculata* (S13 Table and Fig 3). Our assumption is that among the genes belonging to the above-mentioned groups there are at least several that predispose *L*. *seymouri* metabolism to dixeny. Fifty five out of 79 OGs absent in *L*. *pyrrhocoris* do not have any functional annotation assigned and thus represent a broad field for further studies (S13 Table). Nevertheless, several genes identified by comparative genomics approach in our study were already proven to play a pivotal role in parasite survival and virulence (see below).

In order to further narrow down the set of such genes we introduced one more condition into the comparison: gene family present in *L*. *seymouri* must be also present in all *Leishmania* species considered in the analysis (*L*. *braziliensis* MHOM/BR/75/M2903, *L*. *braziliensis* MHOM/BR/75/M2904, *L*. *donovani* BPK282A1, *L*. *infantum* MCAN/ES/98/JPCM5, *L*. *major* MHOM/IL/80/Friedlin, and *L*. *mexicana* MHOM/GT/2001/U1103). A reptile parasite *L*. *tarentolae* ParrotTarII was not included in the analysis due to its inability to infect warm-blooded organisms [46]. Additional BLASTP search (E-value ≤ 10^−10^) for proteins belonging to OGs and meeting the criteria stated above was performed in order to determine whether these OGs have related OGs with homologous proteins clustered separately by the sensitive OrthoMCL algorithm. Cases when related OGs have a presence/absence pattern which violates the abovementioned criteria are not discussed here since unambiguous conclusion cannot be made concerning the role of such proteins in *L*. *seymouri* thermotolerance.

Sixteen OGs absent from *L*. *pyrrhocoris* are shared by *L*. *seymouri* and *Leishmania* spp. Importantly, only 2 of them are absent from both *L*. *pyrrhocoris* and *C*. *fasciculata* (Fig 3). These two OGs represent a kinase-like protein and a ubiquinol-cytochrome *c* reductase-like protein. According to the results of additional BLASTP search, the latter protein OG does not have any related OGs and all of its orthologs in the TriTrypDB are annotated as ubiquinol-cytochrome *c* reductase-like proteins. Aiming to identify homologs of this protein in other species beyond the TriTrypDB, we conducted a BLAST search against the NCBI nr database and found a close homolog only in *Strigomonas culicis* (ubiquinol-cytochrome *c* reductase subunit 6, E-value ≤ 10^−30^, protein accession number: EPY16273.1). The kinase-like protein mentioned above has weak hits with E-value over 10^−30^ to several other OGs containing protein kinases. Due to the relatively high E-values of the BLAST hits and quite unspecific annotations of kinases within related OGs, this protein was not excluded from our analysis (although several related OGs have absence/presence patterns that differ from the required ones), and its possible role in *L*. *seymouri* thermotolerance cannot be ruled out.

Having excluded the requirement of OG being absent from *C*. *fasciculata*, the overlap mentioned above extends to 16 OGs (Fig 3), which include one more group with putative protein kinases as well as putative anaphase-promoting complex subunit, putative epsin and several hypothetical proteins with unknown functions. In order to obtain a global picture of corresponding OG distribution for subunits of the anaphase-promoting complex and putative epsin, we extended analysis of OG presence/absence patterns to the whole dataset of 27 trypanosomatid species. As expected, OGs containing these proteins have shown nearly omnipresent distribution (being absent from *L*. *pyrrhocoris* as required in our analysis and additionally missing in several *Trypanosoma* spp.). Additional BLAST search (with more relaxed parameters) for these proteins against proteins belonging to other OGs also did not return any hits. Such results can be explained assuming considerable sequence diversity in these proteins families. For epsins, a group of eukaryotic proteins broadly implicated in clathrin-mediated endocytosis, there is evidence for substantial sequence dissimilarities and lineage-specific protein architecture [47]. Anaphase-promoting complex is a multi-subunit E3 ubiquitin ligase that is necessary for proteolytic degradation of crucial cell cycle regulators, which causes segregation of sister chromatids [48]. Taking into account a universal role of the proteins mentioned above and their phyletic patterns (especially their presence in several monoxenous species) we conclude that they are unlikely to be involved in *L*. *seymouri* thermotolerance. Interestingly, 3 OGs containing hypothetical proteins (OG_09193, OG_10013, and OG_10042) within the group of 16 OGs fully satisfy the conditions applied in the study, including the absence of closely related OGs. Moreover, these groups of homologous proteins do not occur in any *Trypanosoma* spp. and in monoxenous trypanosomatids for which genome sequences are available (except for *C*. *fasciculata*). Proteins within these groups represent primary targets for additional studies aiming to reveal mechanisms contributing to *L*. *seymouri* thermotolerance.

### *Leptomonas seymouri* harbors dsRNA viruses

**Fig 4:**
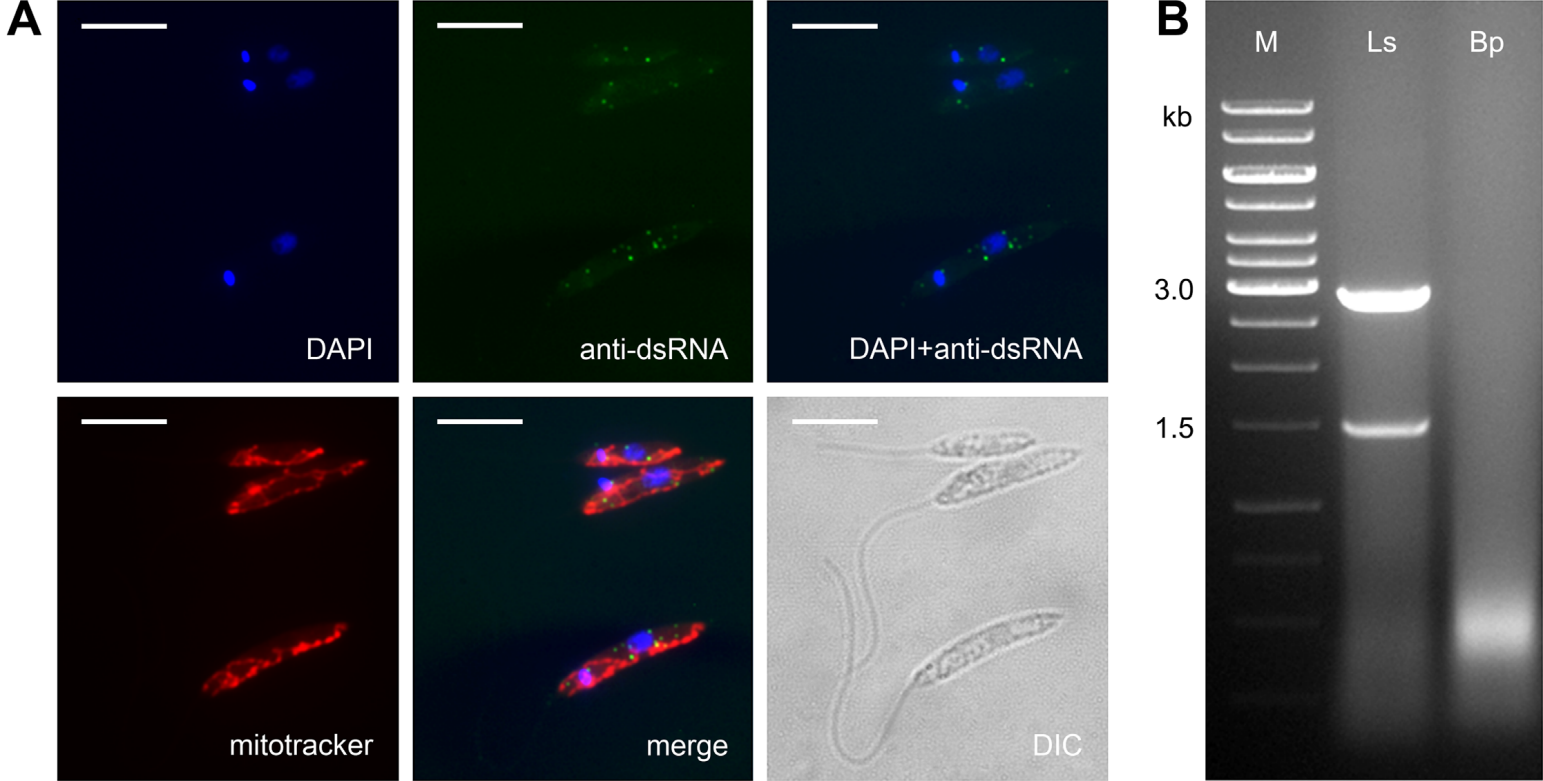
*Leptomonas seymouri* harbors dsRNA viruses. **A**, Cells were stained with DAPI, mitotracker Red and anti-dsRNA antibody for viral detection. Scale bar is 10 μm. **B**, Total RNA samples isolated from *Leptomonas seymouri* (Ls) and *Blechomonas pulexsimulantis* (Bp) were treated with DNase I and S1 nuclease and separated by gel electrophoresis. M– 1 kb ladder (Thermo Fisher Scientific).

Prompted by our observation that *L*. *seymouri* lacks RNAi machinery (see above) and by patterns of RNAi retention in Trypanosomatidae [49], we also examined *L*. *seymouri* for the presence of dsRNA viruses. Two complementary methods, the nuclease digestion assay and immunofluorescence microscopy, were used [50]. Indeed, the anti-dsRNA antibodies detected small sharp dots, which are reminiscent of those found in the virus-positive isolate of *Leishmania guyanensis* [51]. Importantly, these putative viral particles did not co-localize with the mitochondrion (Fig 4A). The nuclease digestion assay of *L*. *seymouri* RNA was performed in parallel with the virus-free *Blechomonas pulexsimulantis* used as a negative control [52]. It detected dsRNA bands resistant to DNase I and S1 nuclease, which were present in RNA preparations from *L*. *seymouri* (Fig 4B). Interestingly, this dsRNAs differ in size from that of the previously characterized LRV1 virus of *Leishmania guyanensis* (1.5 + 2.9 kb versus 5.3 kb, respectively) [53,54]. It remains to be investigated whether this reflects critical differences in genomic organization of viruses, such as segmented versus whole dsRNA genomes.

### Whole transcriptome profiles of *L*. *seymouri* cultivated at different temperatures

To identify genes and/or pathways responsible for thermoresistance of *L*. *seymouri*, we profiled whole transcriptomes of the parasites cultivated at low (23°C) and high (35°C) temperature. We presumed that in addition to genetic factors (e.g. chromosome ploidy) regulation of gene expression may also be involved in adaptation to dixeny. Reads passing the filtering step (61.4; 52.5; 39.1 million reads for replicates at 23°C and 61.1; 58.9; 37.9 million reads for replicates at 35°C) were used in subsequent analyses. Out of 8,488 genes identified in the *L*. *seymouri* genome 8,482 genes were recovered in our analysis. Results of the FDR test are shown in S3 Fig. In total, 340 genes (4% of the total number) were shown to be differentially expressed at the elevated temperature (S14 Table). Expression of 139 genes (1.6% of the total number) was found to be down-regulated at 35°C, whilst 201 genes (2.4% of the total number) were upregulated at least 1.5 fold (p-value ≤ 0.05). Several interesting cases are discussed in detail below.

#### Synthesis of sterols

Sterols and related compounds are important membrane components of the living cells that define cell membrane's fluidity. They act as bidirectional regulators by stabilizing the membrane and raising its melting point at high temperature, and by preventing phospholipids from clustering together and stiffening at low temperature [55]. Sterol biosynthesis is a fairly conserved biochemical pathway in eukaryotes responsible for the production of cholesterol in animals and several C24-alkyl sterols (ergostane-based sterols) in fungi, plants, and trypanosomatids. In *L*. *major*, genetic ablation of C14α-demethylase (C14DM) results in a complete loss of ergostane-based sterols and accumulation of C14-methylated sterols. Genetically modified (c14dm^-/-^) parasites were viable but exhibited some remarkable defects including increased membrane fluidity, and hypersensitivity to heat stress [56]. In *T*. *brucei* the decrease in the levels of squalene synthase and squalene monooxidase led to the depletion of cellular sterol intermediates and end products, impaired cell growth and aberrant morphologies, DNA fragmentation and profound modification of mitochondrial structure and function [57].

In *L*. *seymouri* numerous enzymes implicated in biosynthesis of C24-alkyl sterols were down-regulated at elevated temperature. This list includes sterol C14DM, squalene monooxygenase, lanosterol synthase, C-5 sterol desaturase, and lathosterol oxidase.

#### Oxidative stress protection

As mentioned above, the protection against oxidative stress in monoxenous trypanosomatids is unique since in addition to the trypanothione/ tryparedoxins/peroxiredoxins systems, they heavily rely on a bacterial-type catalase. In *L*. *seymouri*, expression of the trypanothione reductase goes down upon cultivation at 35°C, but this decrease is compensated by overexpression of two other enzymes, namely catalase and ascorbate-dependent peroxidase. The expression of an enzyme responsible for superoxide anions detoxification (superoxide dismutase) is also upregulated at elevated temperature.

#### Carbohydrate and fatty acids metabolism

Several glycosomal components of the carbohydrate metabolism are significantly down-regulated in *L*. *seymouri* at elevated temperature. These are glucose-6-phosphate isomerase, ATP-dependent 6-phospho-1-fructokinase, glycosomal phosphoglycerate kinase, fumarate hydratase, fumarate reductase, and malate dehydrogenases. Conversely, expression of some genes, such as pyruvate phosphate dikinase and cytosolic fumarase, is up-regulated at 35°C.

Consistent with the above-mentioned observations, *L*. *seymouri* catabolism of fatty acids by β-oxidation is enhanced at high temperature. Several enzymes implicated in this reaction, namely enoyl-CoA hydratase, elongase 4, and several desaturases (delta-6 fatty acid desaturase, delta-5 fatty acid desaturase, stearic acid desaturase), are all upregulated at 35°C. Enhanced catabolism is accompanied by diminished *de novo* synthesis, as is evidenced by the inhibition of three consequent elongases (1 to 3) responsible for the synthesis of saturated fatty acids.

### Experimental infection of *Phlebotomus* spp. with *Leptomonas seymouri*

**Fig 5:**
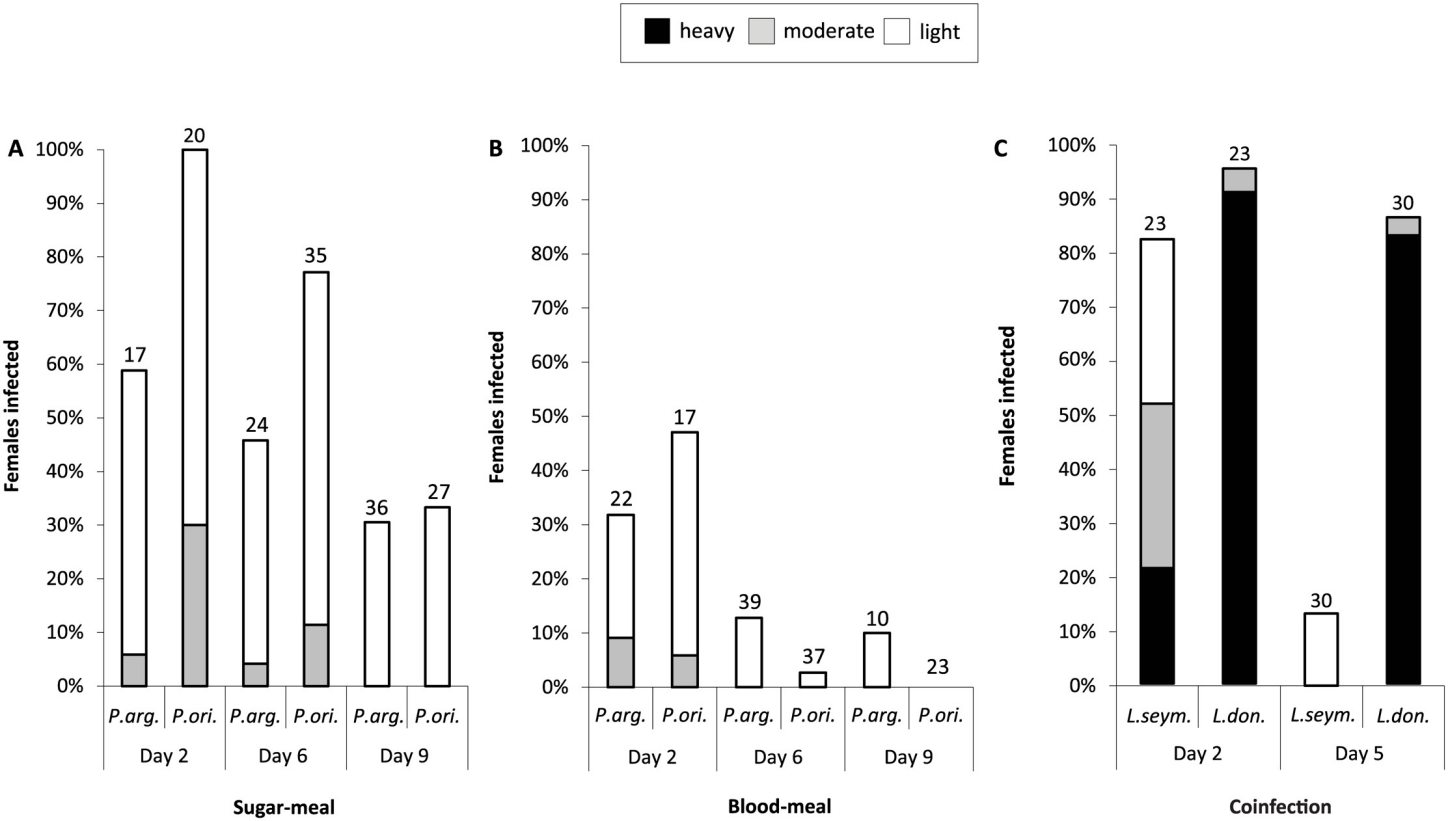
Experimental infection and co-infection of *Phlebotomus* spp. *Phlebotomus argentipes* and *P*. *orientalis* were infected with *Leptomonas*. *seymouri* using sugar- (A) or blood- (B) meal method. Intensity of infection was assayed on days 2, 6, and 9 post infection and defined as light (less than 100 promastigotes, white bar), moderate (100–1,000 promastigotes, grey bar), or heavy (over 1,000 promastigotes, black bar) depending on the number of parasites per gut. **C**, Experimental co-infection of *P*. *argentipes* with mCherry *Leptomonas seymouri* and GFP *Leishmania donovani* GR-374. Intensity of infection was assayed on days 2 and 5 post infection with blood meal and defined as light, moderate, or heavy as above. Numbers above each bar indicate the number of dissected females.

Experimental infections of the two proven vectors of *Leishmania donovani*, *Phlebotomus orientalis* and *P*. *argentipes*, were compared side-by-side. Insects were fed on either blood or sugar meals to mimic the range of conditions which may favor infection (Fig 5A and 5B). On day 2 after infective sugar meal all females of *P*. *orientalis* were infected, while the infection rate of *P*. *argentipes* females was lower (59%) (Fig 5A). Intensity of infection was generally weak in both species tested. On day 6 p. i. percentages of infected sand flies decreased to 77% and 46% for *P*. *orientalis* and *P*. *argentipes* females, respectively. On day 9 every third female remained infected, yet most of them harbored only few flagellates.

Infection *via* the blood meal was less efficient when compared to the sugar meal. On day 2 less than half of blood-fed females of *P*. *orientalis* and *P*. *argentipes* were infected (47% and 32%, respectively). Freely moving promastigotes were found enclosed in the ingested blood. On days 6 and 9 *L*. *seymouri* promastigotes persisted only in a few females (Fig 5B).

### Co-infection of sand flies with *Leptomonas seymouri* and *Leishmania donovani*

The experimental co-infection of sand fly females of *P*. *argentipes* were performed by blood meals containing either mCherry- (*L*. *seymouri*, ATCC-30220) and/or GFP-expressing (*L*. *donovani*, strain GR-374) flagellates. In the control dissection of five sand flies performed just a few hours p. i., both mCherry- and GFP-labeled cells were encountered at about 100 cells of each species *per* sand fly gut. On day 2 p. i. the infection rate of *L*. *seymouri* was lower than that of *L*. *donovani* (82.6% versus 95.7%, respectively), and also the intensity of infection with the former species was significantly weaker (Fig 5C). The differences between both parasite species became even more pronounced on day 5 p. i., when the percentage of infected sand flies remained unaltered for *L*. *donovani* (86.7%), while it markedly dropped for *L*. *seymouri* (13.3%). Moreover, the intensity of infection with *L*. *donovani* was high, whereas the few insects still infected with *L*. *seymouri* harbored only negligible number of free swimming mCherry-expressing cells (Fig 5C).

### Infection of macrophages

Survival of parasites inside mammalian host cells J774 or BMMɸ was evaluated 3, 4, 5 and 6 days p. i. No viable *L*. *seymouri* cells were found in macrophages by fluorescent microscopy or in Giemsa-stained smears. In contrast, the control represented by *L*. *donovani* survived inside both J774 and BMMɸ cells. Similar results were obtained using peritoneal macrophages from BALB/c mice. The transformation assay has confirmed microscopic observations, as no *L*. *seymouri* cells were found after the lysis of macrophages. On the contrary, *L*. *donovani* propagated very well under the same conditions. Similar results were obtained when either J774 or BMMɸ macrophages were simultaneously co-infected with both parasites.

## Discussion

Here we performed a multifarious evaluation of the infective potential of *L*. *seymouri*, repeatedly isolated from kala-azar patients infected by *L*. *donovani* in India and neighboring countries, and have tested the capacity of this monoxenous trypanosomatid to utilize the sand fly vectors permissive for *L*. *donovani*. Moreover, we attempted to find genetic and corresponding metabolic adaptations responsible for its survival at 35°C.

Firstly, we have sequenced the whole genome of *L*. *seymouri* and compared it with *L*. *pyrrhocoris* and *C*. *fasciculata*, the only monoxenous species for which high-quality assemblies are available. Twenty six OGs carried by the thermotolerant *L*. *seymouri* and absent in these closely related thermosensitive flagellates may potentially be associated with this adaptation. Including dixenous species into the comparative analysis narrowed down our search to just two OGs shared by *L*. *seymouri* and five *Leishmania* spp. and absent from *L*. *pyrrhocoris* and *C*. *fasciculata*, namely a kinase-like protein and a ubiquinol-cytochrome *c* reductase-like protein. It was shown previously that protein kinases are involved in amastigote differentiation in *Leishmania* spp. [58], a process in which temperature switch plays a decisive role [59]. Moreover, our search has identified a number of proteins with specific distribution among trypanosomatid lineages (e.g. absent in *Trypanosoma* spp. and/or *Leishmania* spp. but present in monoxenous flagellates) that are prime targets for functional analysis. In any case, the fact that the vast majority of genes within OGs with this phyletic distribution are annotated as hypothetical proteins with unknown function indicates our scarce knowledge of trypanosomatid metabolism.

A number of metabolic changes observed in *L*. *seymouri* exposed to elevated temperature are evocative of those in *Leishmania* amastigotes or *T*. *brucei* bloodstream forms in glucose-poor environment [60,61]. For example, inhibition of the *de novo* synthesis of sterols in *L*. *seymouri* resembles *Leishmania* amastigotes in which the relative abundance of C24-alkyl sterols was significantly decreased upon their differentiation from procyclics [62,63]. Similarly to their dixenous cousins, *L*. *seymouri* cells at high temperature reduce the uptake of glucose and shift their acetyl-CoA production in mitochondria from mainly pyruvate-based to the fatty acids-derived [64–66].

The detection of double-stranded viruses in *L*. *seymouri* is particularly relevant in the light of recent findings that their presence in *Leishmania guyanensis* correlates with its virulence and metastatic potential [51,67]. While molecular mechanisms of this phenomenon are just becoming to be understood, it is already clear that the host immune response is rewired [68,69]. We and others have detected dsRNA-containing viruses in several other monoxenous trypanosomatids parasitizing dipteran and heteropteran insects [27,51,70,71], but their relationships to the characterized viruses of *Leishmania* still remain a mystery. Two analyzed *Leptomonas* spp. differ in their acceptability for dsRNA viruses. This indicates fundamentally different mechanisms they may utilize to regulate their gene expression.

In summary, we conclude that although *L*. *seymouri* has developed several adaptations that allow it to grow well at 35°C, it remains a predominantly monoxenous species not able to infect mammalian macrophages either alone or in co-infection with *Leishmania*. This agrees with a recent report on selective elimination of *Leptomonas* from co-cultures with *Leishmania* [72]. Under certain circumstances it is able to infect mammals, but probably only when the host is immunocompromised by infection with another pathogen, such as *L*. *donovani* or HIV [14,73]. However, it is quite likely that such co-infections are much more frequent than the available literature suggests. This conclusion is further supported by our finding that *L*. *seymouri* can survive up to 9 days in the same sand fly species that is responsible for the transmission of pernicious *Leishmania* spp. Therefore, it will be important to analyze samples from patients suffering from visceral and other leishmaniases with primers specific for *L*. *seymouri* and related (presumably) monoxenous trypanosomatids to address the possibility that we see only the tip of the iceberg. In addition to the capacity to withstand elevated temperature, other factors, such as its ability to escape the host immune response, may likely play an important role in establishment of the *Leptomonas* infection in mammals. We cannot exclude the possibility that some isolates of *L*. *seymouri* may be exclusively transmitted by sandflies and spend part of their life cycle in vertebrates similar to their *Leishmania* spp. relatives.

## Materials and Methods

### Ethics statement

Animals were maintained and handled in the animal facility of Charles University in Prague in accordance with institutional guidelines and Czech legislation (Act Number 246/1992 and 359/2012 coll. on Protection of Animals against Cruelty in present statutes at large), which complies with all relevant European Union and international guidelines for experimental animals. The experiments were approved by the Committee on the Ethics of Animal Experiments of the Charles University in Prague (Permit Number 24/773/08-10001) and were performed under the Certificate of Competency (Registration Number CZU945/05 ext. CZ02573) and the Permission Number 31114/2013-MSMT-13 ext. 24115/2014-MZE-17214 of the Ministry of the Environment of the Czech Republic.

### Origins of strains

*Leptomonas seymouri* isolate ATCC 30220 was obtained from the American Type Culture Collection (ATCC, Manassas, USA). It was isolated from the cotton stainer *Dysdercus suturellus* in the United States in 1959. *Leptomonas pyrrhocoris* isolate H10 [17], *Blechomonas ayalai* isolate B08-376 [52] and *Leishmania donovani* isolate MHOM/ET/2010/GR374 have originated from the research collections at Charles University in Prague, Institute of Parasitology in České Budějovice, and Life Science Research Centre in Ostrava.

### *In vitro* cultivation and morphological analysis

Cultures of the monoxenous trypanosomatids were routinely maintained in the Schneider's Drosophila medium (SDM) (Thermo Fisher Scientific, Waltham, USA) supplemented with 10% Fetal Bovine Serum (FBS) (Thermo Fisher Scientific), 50 units/ml of penicillin, 50 μg/ml of streptomycin (both from Sigma-Aldrich, St. Louis, USA), and 10 μg/ml of hemin (Jena Bioscience GmbH, Jena, Germany) at 23°C. All isolates used in this work can also be cultivated in the Brain Heart Infusion (BHI) medium (Sigma-Aldrich) supplemented with 10% FBS and antibiotics as above, or in the two-phased blood-agar medium [74].

To estimate the dynamics of growth, 5 x 10^4^ parasites were seeded into the SDM or the blood-agar medium. Cultures were incubated at 23°C, 29°C, and 35°C for 7 days. Cell numbers were counted using a hemocytometer and plotted in log scale. Morphology of the cells cultivated at low (23°C) and high (35°C) temperature, either in SDM or blood-agar media, was analyzed at day 4 (exponential phase) after staining cells with Giemsa as described previously [75,76]. One hundred cells per sample were measured and analyzed using ANOVA statistical models [77].

*Leishmania donovani* (MHOM/ET/2010/GR374) transfected with Green Fluorescent Protein (GFP) was cultured in M199 medium (Sigma) containing 20% heat-inactivated FBS (Thermo Fisher Scientific) supplemented with 1% BME vitamins (Sigma), 2% sterile urine, 50 units/ml penicillin, 250μg/ml amikacin (Bristol-Myers Squibb, New York, USA), and 150 μg/ml of geneticin, G418 (Sigma).

### PCR amplification, cloning and sequencing

The internal transcribed spacer, ITS region of the rRNA locus was amplified using primers IAMWE and Tc5.8-rev and conditions described elsewhere [78]. Total genomic DNA samples of clinical Indian kala-azar field isolates Ld_39 and Ld_2001 were used as templates [19]. The 18S rRNA and gGAPDH genes were PCR-amplified, cloned into the pCR2.1 vector system (Thermo Fisher Scientific), sequenced and analyzed as described previously [79,80]. The obtained sequences were deposited to GenBank with the following accession numbers: KP717894, KP717895 (18S rRNA); KP717896, KP717897 (gGAPDH); KP717898, KP717899 (ITS1 + ITS2 regions).

### Genome assembly and annotation

The *Leptomonas seymouri* ATCC 30220 genome was sequenced with 100 nt paired-end reads using the Illumina HiSeq 2000 platform (Macrogen, Seoul, South Korea). Prior to assembly, reads were subjected to trimming and filtering using CLC Genomics Workbench v. 7.0 (CLC Inc, Aarhus, Denmark): regions with Phred quality < 20 were trimmed, no more than one N was allowed in the remaining sequence, then TruSeq adapter trimming and a minimum length threshold of 75 nt were applied.

Draft genome of *L*. *seymouri* was assembled with the CLC Genomics Workbench v. 7.0 employing a De Bruijn graph-based algorithm with the average coverage of 180 x. Augustus v. 2.5.5 was used to annotate the draft genome of *L*. *seymouri* [81]. Prediction accuracy of Augustus was improved by retraining using a training set of *L*. *seymouri* conserved proteins. In brief, *de novo* assembled contigs were searched against proteins in the TriTrypDB v. 7.0 database [82] (BlastX E-value ≤ 10^−5^) and best BLAST hits were chosen based on the following criteria: a) E-value ≤ 10^−30^, b) hit length longer than 80 amino acids (aa), c) percent identity higher than 40. Subsequently, a non-redundant training set of 727 high-confidence gene models with unambiguous start site positions was created based on best BLAST hits to annotated proteins from TriTrypDB and RNA-seq coverage data. Non-redundancy of the training set was achieved by excluding genes with more than 70% identity at the amino acid level. Further analysis of the Augustus annotation included manual curation of predicted genes based on transcriptome sequencing data, e.g. removing start sites predicted in regions with no transcriptomic coverage and adding transcribed ORFs >200 aa in length not predicted by Augustus. For tRNA gene prediction tRNAscan-SE Search Server [83] was used with default parameters. For annotating other non-coding RNAs BlastN algorithm (E-value ≤ 10^−10^) was employed with subsequent manual inspection of BLAST results. As a result, 8,488 genes were annotated in the *L*. *seymouri* genome, which has been submitted to the NCBI (BioProject accession number PRJNA285179) and the TriTryp database, a part of the EuPathDB [84].

### Gene family analysis using the OrthoMCL approach

Orthologous groups are the set of genes descended from a single common ancestral gene, containing both paralogs and orthologs. OGs for *L*. *seymouri* proteins were inferred using the OrthoMCL v.2.0 software [85]. Full proteomes for 23 trypanosomatid species were downloaded from the TriTrypDB v. 7.0 and combined with newly annotated proteins from *L*. *seymouri* and 3 other trypanosomatid species (*Leptomonas pyrrhocoris*, *Blechomonas ayalai* and *Paratrypanosoma confusum*). The reference protein dataset was subjected to removal of poor quality proteins (based on sequence length and percent of in-frame stop codons), all vs. all BLAST (E-value 10^−10^) and a clustering procedure implemented in the OrthoMCL algorithm. This resulted in 19,866 OGs, 7,935 of which contained proteins of *L*. *seymouri*.

### Whole transcriptome data processing and analysis

*L*. *seymouri* was cultivated at 23°C and 35°C for 75 hrs. Total RNA was isolated from 2.5 x 10^7^ cells using RNeasy Mini Kit (Qiagen GmbH, Hilden, Germany) according to the manufacturer’s instruction. The mRNA-derived libraries were sequenced with 100 nt paired-end reads on the Illumina HiSeq 2000 platform (Macrogen). Total of 3 independent biological replicates were analyzed. The whole transcriptome data from this study have been submitted to TriTrypDB database [82].

Differential gene expression analysis was done using the RNA-Seq tool in CLC Genomics Workbench. Raw reads were subjected to quality-based trimming (regions with Phred quality < 20 were trimmed, no more than one N was allowed in the remaining sequence), adapter trimming, and a minimum length threshold of 30 bp. Processed reads were then mapped to the annotated *L*. *seymouri* genome with the following parameters: maximum number of mismatches, 2; minimum fraction of read length mapped, 0.8; minimum identity within the mapped sequence, 0.8; maximum number of best-scoring hits for a read, 30. All libraries were mapped as paired-end, and expression values (RPKM) for each gene were calculated. To identify gene sets that are differentially expressed between the two conditions, the FDR test was employed [86]. Genes with expression fold change ≥ 1.5 and FDR p-value ≤ 0.05 were chosen for further analyses.

Gene ontology (GO) terms for genes up- and down-regulated at high temperature were generated using the Blast2GO plugin in CLC Genomics Workbench [87]. Initially, BlastP search against the NCBI nr database was performed, GO terms associated with all the hits were retrieved, and most appropriate GO terms were selected according to the standard Blast2GO procedure. GO term enrichment was assessed using Fisher's exact test.

### Detection of dsRNA viruses

For detection of dsRNA viruses, two complementary protocols were used. Cells were stained with 4′,6-diamidino-2-phenylindole (DAPI), mitotracker Red CMXRos (both from Thermo Fisher Scientific) and mouse monoclonal anti-dsRNA antibody (Scicons, Szirák, Hungary), followed by goat anti-mouse IgG–Alexa Fluor 488 (Thermo Fisher Scientific) antibody as described previously [50]. In addition, 50 μg of total RNA isolated using TRI reagent (Sigma-Aldrich) was treated with 1 unit of DNase I (New England Biolabs, Ipswich, USA) at 37°C for 1 hr, followed by digestion with 35 units of S1 nuclease (Sigma-Aldrich) for 45 min at the same temperature. Samples were analyzed on 0.8% native agarose in 1xTAE buffer [88].

### Establishing a fluorescent strain of *L*. *seymouri*

A fragment encoding mCherry fluorescent protein was amplified with primers 5´-TTATCCATGGTTAGTAAAGGAGAA-3´ and 5´-TGTTAGCGGCCGCTTATGCGGTACCAGAACC-3´ using plasmid p2686 as a template [89]. The resulting 745 bp fragment was cloned into the pF4T7polNLS1.4sat vector digested with *Nco*I and *Not*I replacing the T7 polymerase ORF [90]. Log-phase *L*. *seymouri* cells (4 x 10^7^) were transfected with 15 μg of *Swa*I-linearized pF4mCherry1.4sat as described before [91]. Recombinant clones were selected on agar—BHI growth medium supplemented with 10% FBS, 40mM HEPES, pH 7.4 and nourseothricin (Jena Bioscience) at final concentration of 250 μg/ml. Expression of mCherry was confirmed by fluorescence microscopy.

### *L*. *seymouri* development in sandflies

Colonies of two sand fly species, *Phlebotomus orientalis* and *P*. *argentipes*, both representing major proven vectors for *L*. *donovani*, were maintained under standard conditions as described elsewhere [92]. Females of both colonies were fed either through a chick-skin membrane on suspension of heat-inactivated rabbit blood containing exponentially growing 1 x 10^7^ promastigotes per ml of blood or on 20% sucrose solution containing 5 x 10^7^ promastigotes per ml. In order to recognize sugar-fed females, the sucrose solution was stained by indigo carmin. Blood- and sugar-fed females were kept at 26°C with free access to 50% sucrose solution by day 1 post infection (p. i.).

Sand fly females were dissected at different intervals p. i. (1–2, 5–6 and 7–9 days). Numbers and location of flagellates in the sand fly gut were checked microscopically. Parasite loads were graded as previously described, i.e.: light (< 100 parasites/gut), moderate (100–1,000 parasites/gut) and heavy (> 1,000 parasites/gut) [93].

### *Leptomonas* and *Leishmania* co-infection and development in sand flies

Females of *P*. *argentipes* were fed through a chick-skin membrane on suspension of heat-inactivated rabbit blood containing 1 x 10^6^ per ml promastigotes of *Leptomonas seymouri* mCherry (passage 4) and 1 x 10^6^ per ml promastigotes of *Leishmania donovani* (MHOM/ET/2010/GR374) GFP (passage 10) originating from exponentially growing cultures. Assorted blood-fed females were kept at 26°C with free access to 20% sucrose solution. Sand fly females were dissected on days 2 and 5 p. i., and the presence of parasites as well as other characteristics were analyzed as described previously [93].

### *In vitro* infection of mouse macrophages with *Leptomonas seymouri* and *Leishmania donovani*

Macrophage cell line J774 was cultured in complete RPMI-1640 medium (Sigma) containing 10% FBS, 100 U/ml of penicillin, 100 μg/ml of streptomycin, 2mM of L-glutamine, and 0.05 mM of β-mercapto-ethanol (all from Sigma) at 37°C with 5% CO_2_. Bone marrow was obtained by flushing of tibias and femurs of BALB/c mice and flagellates were cultured in complete RPMI-1640 medium (Sigma) supplemented as above along with 20% of L929 fibroblast cell culture supernatant serving as a source of macrophage colony-stimulating factor at 37°C with 5% CO_2_. The differentiation from bone marrow precursor cells to bone marrow-derived macrophages proceeded for 7 to 8 days in sterile polystyrene Petri dishes. The bone marrow derived macrophages (BMMɸ) were washed and seeded into plates at density of 5 x 10^5^ cells per ml. Consequently, stationary cultures of *Leishmania donovani* (GFP), *Leptomonas seymouri* (mCherry), alone or in combination were added in ratio of 8:1 (parasites: BMMɸ). Three days p. i. BMMɸ were extensively washed with pre-warmed RPMI-1640 to remove excess of parasites and the viability of trypanosomatids was monitored by fluorescence microscope Olympus CX-31 (Olympus, Tokyo, Japan) up to 6 day p. i. In addition, Giemsa staining was used to analyze intracellular forms in macrophages by light microscopy. All experiments were performed in two independent biological replicates.

To analyze survival of parasites, the transformation growth assay was used [94]. In brief, macrophages infected with *Leishmania donovani*, *Leptomonas seymouri*, alone or in combination for 96 hrs were extensively washed with RPMI-1640 and lysed with 0.016% SDS in RPMI-1640 for 7 min at room temperature to release their intracellular forms. The lysis reactions were neutralized by RPMI-1640 supplemented with 17% heat-inactivated FBS. Parasites were spun down at 3,200 rpm for 10 min at 4°C, washed in RPMI-1640, and re-suspended in a relevant promastigote medium (BHI or M199) supplemented with an appropriate selective antibiotic at 23°C. For macrophages co-infected with both parasites, two types of media and antibiotics were assessed. The status of viable parasites was checked for 6 consecutive days.

## Supporting Information

S1 Fig Evolutionary relationships among trypanosomatids based on SSU rRNA sequences. Green color depicts bodonids species used as an outgroup. Yellow and red colors represent monoxenous and dixenous parasites, respectively. Clade Leishmaniinae is marked. Modified from [28].
(EPS)

S2 Fig Cladogram showing phylogenetic relationships among the members of sub-family Leishmaniinae. (EPS)

S3 Fig Volcano plot of the *L*. *seymouri* genes differentially expressed at low (23°C) and high (35°C) temperature. Positive and negative Δlog2RPKM values indicate genes up- and downregulated, respectively. Statistically significant (FDR p-value ≤ 0.05) up- and downregulated (with a 1.5-fold threshold) genes are boxed.
(EPS)

S1 Table GenBank accession numbers of *L*. *seymouri* sequences misidentified as *Leishmania* spp. (XLSX)

S2 Table List of genes involved in glycolysis. (XLSX)

S3 Table List of genes involved in carbohydrate metabolism. (XLSX)

S4 Table List of genes involved in amino acid metabolism. (XLSX)

S5 Table List of genes of mitochondrial origin. (XLSX)

S6 Table List of genes involved in fatty acid biogenesis. (XLSX)

S7 Table List of genes involved in urea cycle. (XLSX)

S8 Table List of genes encoding surface proteins. (XLSX)

S9 Table List of genes involved in oxidative stress response. (XLSX)

S10 Table List of genes involved in RNA interference. (XLSX)

S11 Table List of genes acquired by lateral transfer. (XLSX)

S12 Table Proteome datasets used in the OrthoMCl analysis. (XLSX)

S13 Table List of all 79 OGs analyzed in the study. (XLSX)

S14 Table List of genes differentially expressed at low (23°C) and high (35°C) temperature. (XLSX)
